# Non-24-Hour Sleep-Wake Disorder Revisited – A Case Study

**DOI:** 10.3389/fneur.2016.00017

**Published:** 2016-02-29

**Authors:** Corrado Garbazza, Vivien Bromundt, Anne Eckert, Daniel P. Brunner, Fides Meier, Sandra Hackethal, Christian Cajochen

**Affiliations:** ^1^Centre for Chronobiology, Psychiatric Hospital of the University of Basel, Basel, Switzerland; ^2^Transfaculty Research Platform Molecular and Cognitive Neurosciences, University of Basel, Basel, Switzerland; ^3^Sleep-Wake-Epilepsy-Centre, Department of Neurology, Inselspital, Bern University Hospital, Bern, Switzerland; ^4^Neurobiology Laboratory for Brain Aging and Mental Health, Psychiatric Hospital of the University of Basel, Basel, Switzerland; ^5^Center for Sleep Medicine, Hirslanden Clinic Zurich, Zurich, Switzerland; ^6^Charité – Universitaetsmedizin Berlin, Berlin, Germany

**Keywords:** non-24-hour sleep-wake disorder, circadian rhythm sleep disorders, bright light therapy, melatonin, Hodgkin’s lymphoma

## Abstract

The human sleep-wake cycle is governed by two major factors: a homeostatic hourglass process (process S), which rises linearly during the day, and a circadian process C, which determines the timing of sleep in a ~24-h rhythm in accordance to the external light–dark (LD) cycle. While both individual processes are fairly well characterized, the exact nature of their interaction remains unclear. The circadian rhythm is generated by the suprachiasmatic nucleus (“master clock”) of the anterior hypothalamus, through cell-autonomous feedback loops of DNA transcription and translation. While the phase length (tau) of the cycle is relatively stable and genetically determined, the phase of the clock is reset by external stimuli (“zeitgebers”), the most important being the LD cycle. Misalignments of the internal rhythm with the LD cycle can lead to various somatic complaints and to the development of circadian rhythm sleep disorders (CRSD). Non-24-hour sleep-wake disorders (N24HSWD) is a CRSD affecting up to 50% of totally blind patients and characterized by the inability to maintain a stable entrainment of the typically long circadian rhythm (tau > 24.5 h) to the LD cycle. The disease is rare in sighted individuals and the pathophysiology less well understood. Here, we present the case of a 40-year-old sighted male, who developed a misalignment of the internal clock with the external LD cycle following the treatment for Hodgkin’s lymphoma (ABVD regimen, four cycles and AVD regimen, four cycles). A thorough clinical assessment, including actigraphy, melatonin profiles and polysomnography led to the diagnosis of non-24-hour sleep-wake disorders (N24HSWD) with a free-running rhythm of tau = 25.27 h. A therapeutic intervention with bright light therapy (30 min, 10,000 lux) in the morning and melatonin administration (0.5–0.75 mg) in the evening failed to entrain the free-running rhythm, although a longer treatment duration and more intense therapy might have been successful. The sudden onset and close timely connection led us to hypothesize that the chemotherapy might have caused a mutation of the molecular clock components leading to the observed elongation of the circadian period.

## Introduction

In summer 2012, a 40-year-old sighted male patient was referred to our center, because of a suspected misalignment between his biological clock and the external light–dark (LD) cycle. He reported to suffer from daily lapses of sleep on- and offset with a self-calculated free-running circadian rhythm of more than 25 h. The trained electro-engineer presented with an unusual set of circumstances preceding the onset of the symptoms: in 2009, he was diagnosed with Hodgkin’s lymphoma (Stage IVA). He was treated with a standardized regimen of multiple courses of polychemotherapy (ABVD regimen: adriamycin 50 mg/bleomycin 20 mg/vinblastine 10 mg/dacarbazine 750 mg; four cycles ABVD, four cycles AVD). Luckily, the patient responded well and is still in full remission. However, during the oncological treatment, he progressively began suffering from tiredness and inattentiveness during the day and noticed a gradual shift of his sleep-wake rhythm to later hours. After completing the treatment, he remained unemployed and could therefore freely organize his daily schedule according to his preferred activity and sleeping times, which he managed to do using a calendar app on his smartphone. At the time of the consultation, he had experienced a free-running rhythm for more than 3 years, did not suffer from any social disability arising from the symptoms, and reported physical and psychological wellbeing, apart from a difficulty in concentrating and a lack of attention during wakefulness.

Subjective measures of the patient’s daytime sleepiness were assessed using the Epworth Sleepiness Scale (ESS) (1) and the Pittsburgh Sleep Quality Index (PSQI) (2), which both did not show any pathological findings (ESS = 0/24, PSQI daytime sleepiness – subscore = 2/3). In particular, the ESS score was 0 of 24 points (pathological > 10 points); however, the patient remarked to actually experience fatigue with inattentiveness and lack of concentration, rather than actual daytime sleepiness.

To quantify the described symptoms, we conducted multiple standard tests used for the assessment of circadian sleep-wake disorders, including non-dominant wrist actigraphy recordings with activity storage in 1-min intervals over 5 months (Actiwatch, Cambridge Neurotechnology Ltd., UK), serial melatonin measurements in saliva samples collected in 1–3 h intervals across 24–48 h (Salivettes, Sarstedt AG, Switzerland; direct double-antibody immunoassay, sensitivity of 0.2 pg/ml, Bühlmann Laboratories AG, Allschwil/Switzerland), an in-lab melatonin suppression test by bright light (Daylight, Uplift Technologies, Canada; 10,000 lux measured on eyelevel, 20 cm distance to the light source), and polysomnography (PSG; Vitaport-3 digital recorder and Vitaport sleep scoring software, TEMEC Instruments BV, Kerkrade, Netherlands).

We also analyzed the patient’s circadian rhythm at the molecular level by measuring the circadian period (tau) of clock gene transcription in cultivated fibroblasts isolated from five skin biopsies. In this technique, a mouse *Bmal1* promoter, which drives the expression of a luciferase gene, is introduced into the fibroblasts by lentiviral transfection. After synchronization with dexamethasone, tau is calculated from the circadian bioluminescence of the cell cultures. The *in vitro* analysis was conducted in every detail as described in the work of Pagani and colleagues, to which we refer for further information (3).

**Figure 1:**
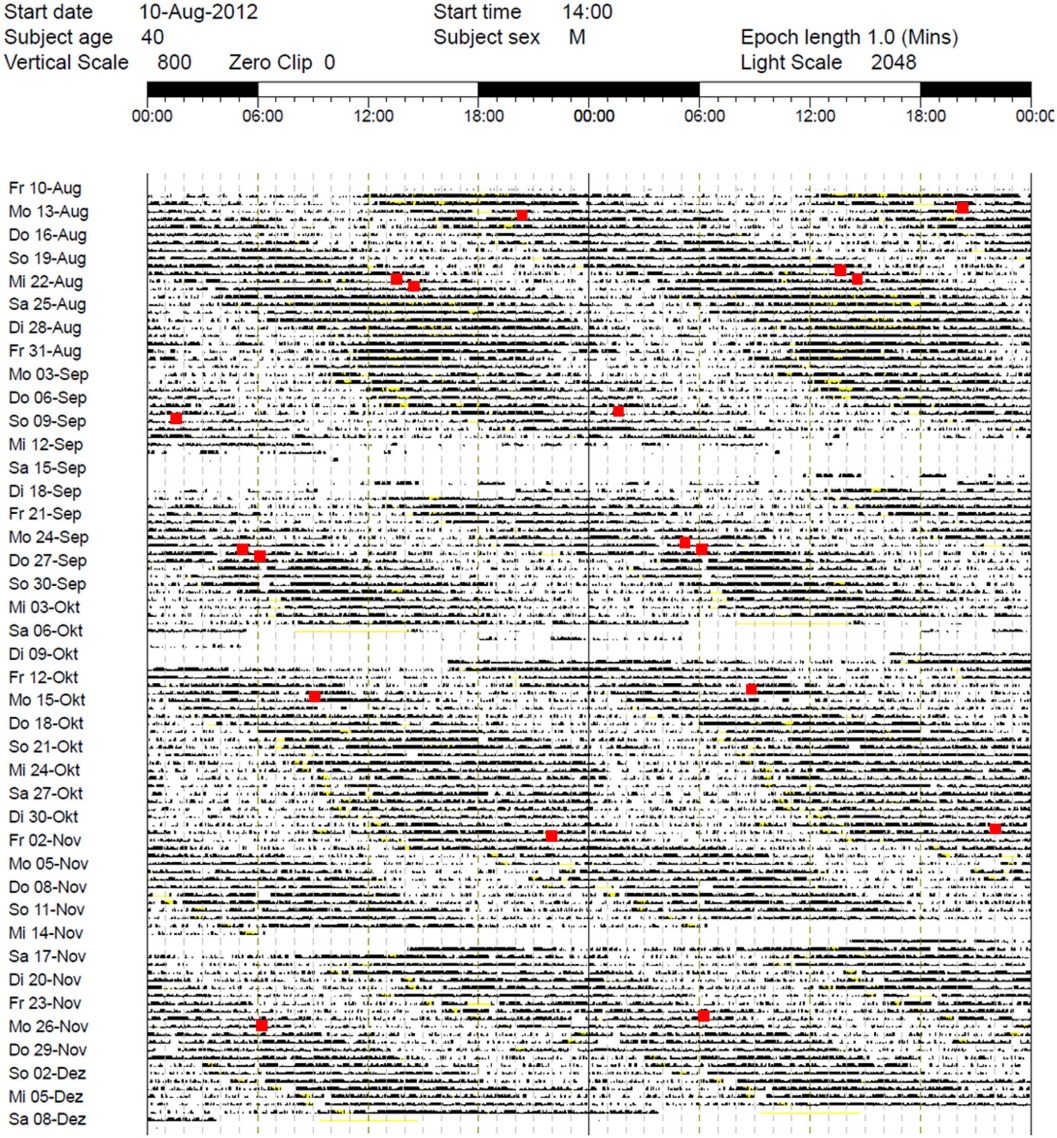
**Actigraphy recording over 5 months (August–December 2012)**. Note the free-running rhythm of activity (black bars) and sleep-periods (lighter areas) with a calculated intrinsic period of tau = 25.27 h and a mean phase angle of 3.38 ± 2.27 h between dim light melatonin onset (DLMO) and sleep onset. Red spots: DLMO = 3 pg. The patient received 30 min BLT in the morning between October 21, 2012 and December 7, 2012 and oral melatonin in the evening from October 21, 2012 to October 24, 2012 (discontinued due to side effects). During the first days of treatment, sleep onset continued to free-run, while sleep offset remained relatively stable (for ~10 days).

**Figure 2:**
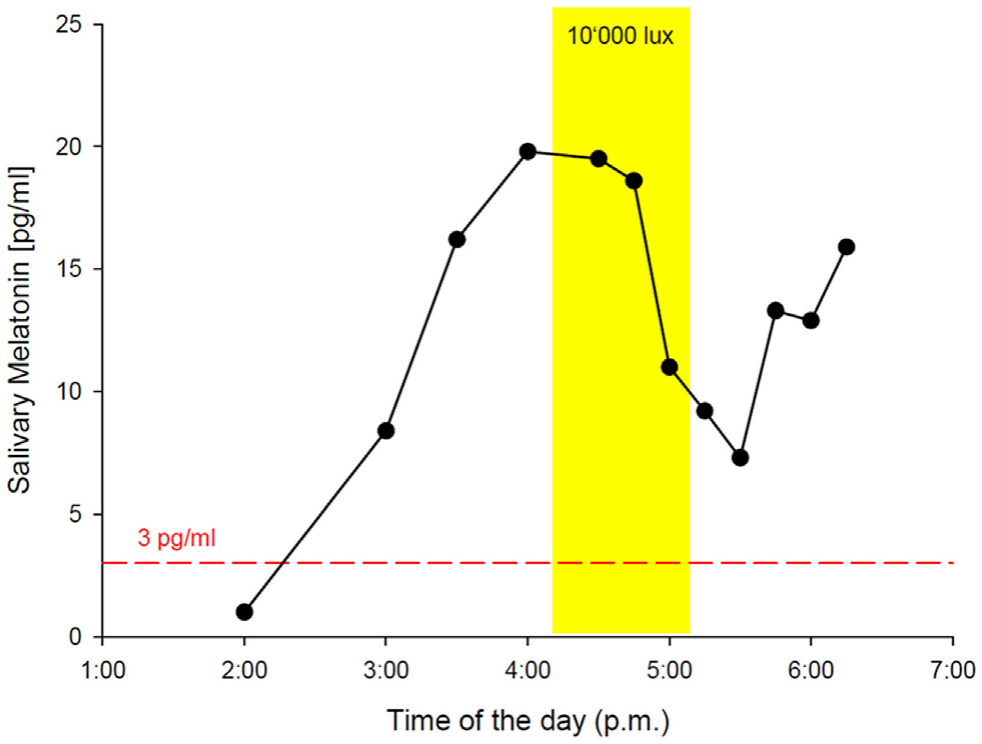
**In-lab melatonin suppression test with bright light (BL: 10’000 lux)**. Schedule: the patient rose at 2:45 a.m. (according to his free-running rhythm), came to our lab at 10 a.m. and was kept under dim light conditions (<8 lux). Saliva samples for melatonin measurement were collected hourly from 11:00 a.m. on. DLMO (3 pg threshold, red dashed line) occurred at 2:16 p.m. The melatonin suppression test was scheduled from 4:30 p.m. to 5:39 p.m. with saliva samples in 15 min intervals. BL (yellow rectangle) caused a significant reduction of melatonin secretion, indicating a normal physiological response *via* an intact retina–RHT–pineal axis. The patient spent the following night in the lab for PSG recording with sleep onset at 6:40 p.m. and sleep offset at 3:45 a.m.

While the results from the PSG were within the normal range (five NREM-REM cycles with slightly increased fragmentation due to short wake periods, sleep efficiency = 82% at 9 h time in bed, and normal proportions of sleep stages), the actigraphy recording showed a free-running sleep-wake rhythm with a phase length (tau) of 25.27 h (Figure 1). The melatonin profiles showed a similar free-running rhythm, synchronous to the observed sleep-wake cycle, however, with a prolonged mean phase angle of 3.38 ± 2.27 h between melatonin onset and sleep onset. The melatonin suppression test by bright light (10,000 lux, see Figure 2) showed a normal response of the physiological LD-mediated melatonin release from the pineal gland. The analysis of tau in fibroblasts confirmed our *in vivo* measurement and showed an even longer tau *in vitro* (=25.6 h) than *in vivo*.

Based on the collected data, we confirmed the diagnosis of N24HSWD and decided for a therapeutic approach based on melatonin administration (0.5–0.75 mg) in the evening, combined with bright light therapy (10,000 lux for 30 min) in the morning.

The combination of melatonin, morning light therapy, and regular wake-up times (set with an alarm clock) during the first week of treatment led to an apparent stabilization of sleep offset in the morning. However, sleep onset still followed the original free-running rhythm with consecutive delays every day (Figure 1). The profile of melatonin secretion was also unaffected by the treatment and remained non-entrained. Unfortunately, the patient had to stop taking melatonin after only 4 days because it triggered severe headaches.

In summary, the applied therapeutic intervention was unable to resynchronize the free-running rhythm of the patient. Nevertheless, he decided to continue with morning light therapy after getting up, because he experienced improved attentiveness and concentration during wakefulness.

## Background – The Molecular Clock

The ability to anticipate predictable environmental changes and adapt behavioral responses to daily or seasonal variations is a highly conserved biological program among various species, from single cell organisms to more complex life forms, such as insects, rodents, birds, and humans (4).

The phenomenon’s underlying principle, a genetically encoded “molecular clock,” basically consists of positive and negative feedback mechanisms of DNA translation: the dimerization of the transcription factor CLOCK with BMAL1/2 or NPAS2 initiates the expression of the clock proteins PERIOD (PER1, PER2, and PER3) and CRYPTOCHROME (CRY1 and CRY2), which accumulate and inhibit CLOCK:BMAL1/2 (or CLOCK:NPAS2) activity, blocking their own expression and therefore closing the cycle (5). These molecular loops intrinsically generate a circadian rhythm (lat. *circa diem* = about a day), which is then directly or indirectly translated into behaviors, such as locomotion and feeding or sleep-wake cycles, according to environmental settings. While the period length (tau) remains relatively stable, the phase is influenced and reset by external cues, with the most important “zeitgeber” being the 24-h LD cycle (6).

In mammals, the circadian timing system is organized in a hierarchical manner, with the suprachiasmatic nucleus (SCN), a formation of 20,000 neurons in the anterior hypothalamus, acting as the “master clock” (7). These clock neurons directly receive non-visual light information from photoreceptive retinal ganglion cells (RGCs) (6, 8) *via* the retinohypothalamic tract, resetting and synchronizing their internal rhythm to the environmental LD cycle (6, 9). This self-sustained neuronal oscillator then disseminates the integrated circadian information through direct or indirect electrical and humoral pathways to “slave oscillators” in peripheral tissues, what ultimately leads to the circadian expression of behavior (10, 11).

The systems’ plasticity and ability to incorporate external information is vital for reacting to changes of the environment but makes it also vulnerable to non-physiological cues introduced by our modern day life, such as the light exposure during the evening hours, shift work, or the crossing of multiple time zones by transmeridian flights. These conditions can cause a misalignment of the internal and external phase, resulting in circadian rhythm sleep disorders (CRSD), such as shift work sleep disorder (SWSD), advanced sleep phase syndrome (ASPS), delayed sleep phase syndrome (DSPS), jet lag (JL), and non-24-hour sleep-wake disorder (N24HSWD) (12). The commonality of CRSD is the general inability to fall asleep or rise at the desired time of the day due to asynchrony of the internal clock with the external LD cycle, leading to daytime sleepiness with lack of concentration, social dysfunction, and a predisposition to various clinical conditions ranging from metabolic disorders to cancer (13–15).

## Non-24-Hour Sleep-Wake Disorder

**Figure 3:**
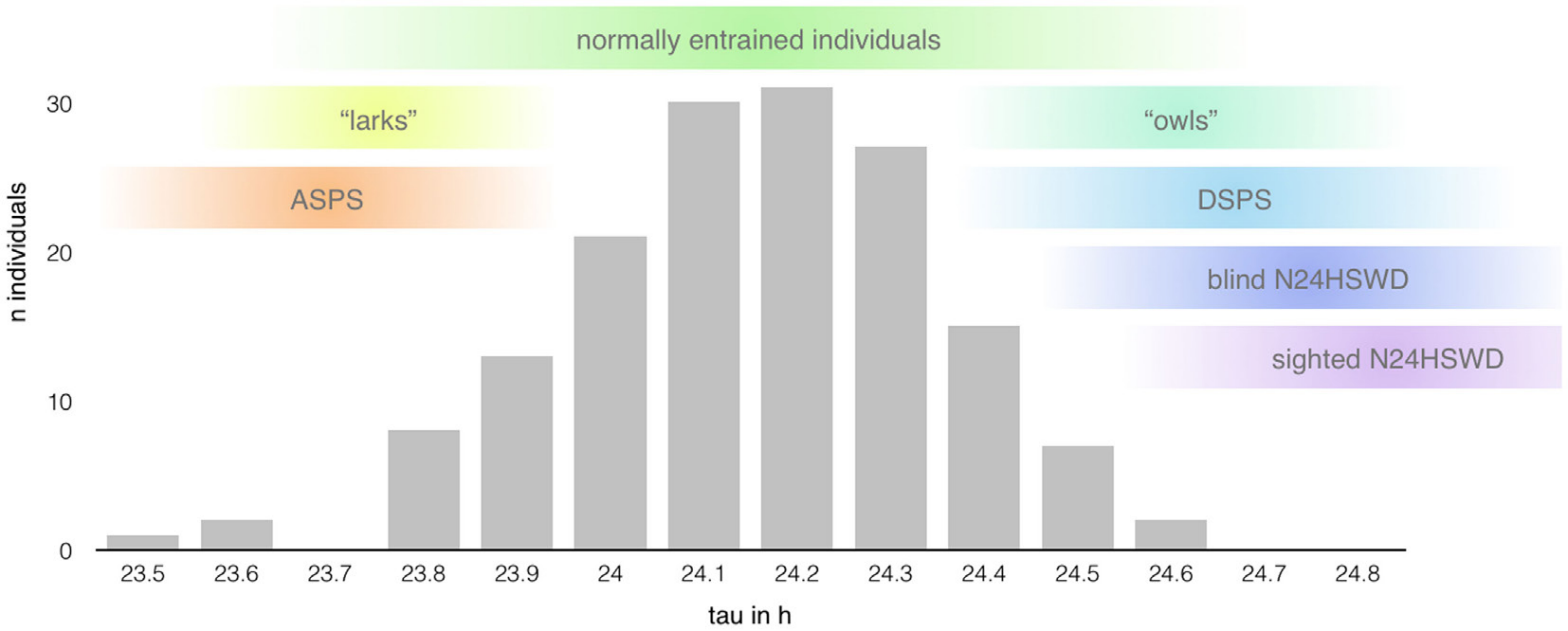
**Range of the intrinsic period length (based on data presented in Ref. (14); *n* = 157 healthy individuals)**. The length of the circadian period shows a rather large interindividual variability mimicking a bell distribution centered around 24.15 h (±0.2 h) with extreme taus predisposing to the development of certain circadian rhythm disorders (12). However, the range of normal entrainment, as well as pathological alignment, seems to show rather large overlaps in the length of tau, indicating the influence of other factors in the determination of chronotypes and the development of circadian rhythm sleep disorders, respectively.

The internal period length (tau – τ) of individuals with normal sleep is in average slightly longer than the environmental LD cycle (about 24.15 ± 0.2 h, see Figure 3) with a shorter average tau in women (24.09 ± 0.2 h) than in men (24.19 ± 0.2 h) (16). Daily entrainment of the SCN by light exposure and other phase-shifting agents ensures the alignment through the shift of the internal rhythm.

In N24HSWD the affected individuals typically show an intrinsic rhythm longer than 24 h and the inability to maintain a stable entrainment to the external LD cycle. This results in a daily gradual shift of sleep on- and offset and consequently causes the behavioral rhythms to free-run. Therefore, affected patients show a cycling, relapsing–remitting pattern of sleep disturbances, with insomnia and/or excessive daytime sleepiness, fatigue, difficulty concentrating, and memory problems in the symptomatic phases, when the internal rhythm is out of sync with the solar day, and completely asymptomatic periods (13, 17). The direct negative influences on social activities and work obligations might contribute to a worsening of the quality of life and to the development of psychiatric illnesses, especially affective disorders (13, 17).

Most patients affected by N24HSWD are totally blind individuals, with an estimated prevalence of ~50% or more in this population (13, 18). The average period length of ~24.5 h (range 23.8–25.1 h) in these subjects shows an overlap with the range of tau of normally entrained individuals (12), but the lack of a functional pathway of light transmission (depending on the cause of the blindness) disables the regulating role of light as “zeitgeber” and therefore the entrainment of the circadian pacemaker to the 24-h day (17).

In contrast, N24HSWD in sighted individuals is a rare condition with symptom onset in teenage years and preference for the male sex (4:1 ratio). Affected individuals often show an extreme period length ranging from 24.5 to 25.5 h or more (17), which is thought to exceed the capability of entrainment, making it the primary risk factor for the development of this disorder. Other likely contributing factors are a decreased or heightened response of the clock mechanism to light, reduced or untimely environmental or social cues (in about a quarter of patients due to comorbid psychiatric disorders), and genetic variation of alleles of the molecular clock, although no specific variant has been directly linked to N24HSWD yet (17). However, the exact mechanism and extend of contribution of each presumed pathophysiological aspect remain unclear.

The clinical assessment of individuals with documented sleep problems related to an abnormal synchronization between the 24-h LD cycle and the endogenous circadian rhythm consists of at least two consecutive weeks of actigraphy with sleep diaries and continuous measurement of the core body temperature or serial measurement of melatonin in serum, saliva, or urine (ICSD-3) (17, 19).

Once diagnosed, the therapeutic aim is to resynchronize the longer than 24 h sleep-wake cycle to the 24-h day by inducing a daily shift of the circadian pacemaker.

Although there is insufficient evidence supporting the effectiveness of any treatment option for sighted individuals with N24HSWD to date (20), experts suggest the use of bright light therapy and/or melatonin administration (21).

In sighted patients, morning bright light should be applied when their circadian phase is synchronous to the solar day in order to stop a further shift to later hours. Melatonin is given in the evening (in blind and sighted individuals) to promote sleep onset at proper clock times and avoid a progressive phase delay due to the free-running rhythm (21). Furthermore, a new melatonin receptor agonist (22), which preferably acts on the MT2 receptor (responsible for the phase-shifting abilities of melatonin), recently gained EMA approval for the treatment of N24HSWD in blind patients (23).

## Discussion

Over the course of the past decades, several case reports about sighted patients suffering from N24HSWD have been published, but only a small number refers to patients with symptom onset directly related or attributed to external events, the most common being head trauma (24, 25). The presented case combines several possible triggers of N24HSWD in our patient, each of which theoretically may lead to unfavorable light exposure; lacking social engagement; new habits concerning sleep, exercise, and meal times; and the development of affective disorders.

**Figure 4:**
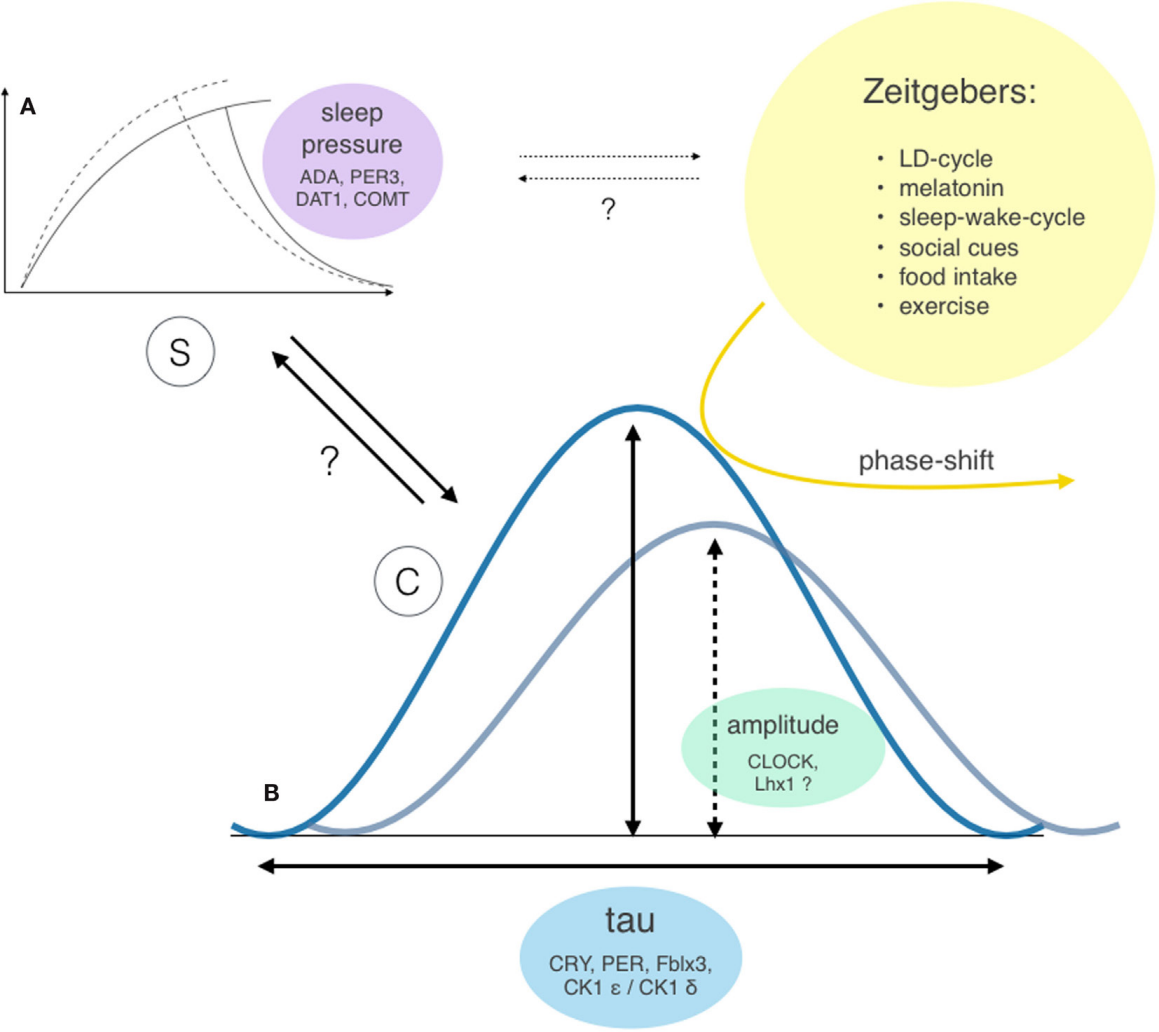
**Two-factor model of sleep-wake regulation with interactions and genetic modifiers of the involved factors**. Sleep regulation is governed by two major factors: the homeostatic process S **(A)** and the circadian process C **(B)** (26, 27). However, the exact interaction between these two factors (bold arrows) and individual extend of contribution to the sleep-wake regulation remains unclear. Both processes show considerable interindividual differences: the ability to build up sleep pressure and therefore vulnerability to sleep loss was shown to depend on gene polymorphism of enzymes involved in dopamine and adenosine processing (29, 30) and polymorphisms of the clock gene PER3 (31). The circadian rhythm incorporates two different properties: the bell-distributed, genetically determined cycle length *tau* and the amplitude of the expressed oscillation [e.g., Bmal1 transcription (33), which influences the vulnerability of the system to resetting stimuli (yellow arrow)]. Potential further interactions between process S and external zeitgebers are indicated as dotted arrows.

Sleep-wake regulation is governed by two major processes (26, 27) – the homeostatic sleep drive (process S), which linearly rises during waking hours, and the circadian rhythm (process C), which modulates the timing of sleep – and shows a high level of interindividual variability. An individual combination of different gene polymorphisms directly or indirectly influences the involved parameters (see Figure 4), such as the circadian rhythm itself (28), the homeostatic sleep drive (29–31), as well as the systems’ ability to receive, process, and integrate external zeitgebers (32).

As mentioned above, the greatest risk factor for the development of “endogenous” CRSD (12) is the length of the circadian cycle. However, the relationship between the intrinsic period length and the development of CRSD seems to be not that linear (see Figure 3). Tau of normally entrained individuals, different chronotypes, and patients suffering from CRSD shows significant overlaps (34), suggesting additional factors at play.

In this case, the close timely connection between symptom onset and the cancer treatment could lead the argument in two possible directions: first of all, since the patients’ endogenous cycle length prior to the treatment is unknown, it is possible that he always had a rather long tau and the loss of a structured daily life just uncovered the free-running rhythm. One could even argue that the chronic misalignment of his internal tau with the external LD cycle in the sense of a “social jet lag” predisposed him to the development of the lymphoma in the first place – epidemiological studies point to a close relationship between alterations of core clock genes as well as chronic circadian misalignment (e.g., by shift work) and susceptibility to cancer (14, 15).

On the other hand, the aggressive chemotherapy regimen used to treat lymphoma is in 20% of the cases associated with the development of secondary cancers caused by specific gene mutations (35). Topoisomerase II inhibitors, such as adriamycin, are known to cause chromosomal rearrangements involving the *MLL* gene, which encodes histone-modifying enzymes linked to transcriptional activation. *MLL1*, a H3K4-specific methyltransferase, was shown to directly interact with CLOCK:BMAL1 and contribute to its rhythmic recruitment to circadian promotors, facilitating circadian gene transcription (36). Another member of the family, *MLL3*, was also shown to play a fundamental role in gene transcription of the core clock mechanism itself: mouse liver cells containing catalytic inactive *MLL3* showed severe disruption of *PER1/2, CRY1/2*, and *Bmal1* transcription (37). A modification of the MLL gene by the chemotherapy could, therefore, potentially have caused a disruption of circadian gene transcription, contributing to the observed extreme phenotype.

In general, especially alterations within the negative feedback loop seem to modify the cycle length. The fine-tuning mechanism extending tau to ~24 h involves the post-translational modification of CRY1, CRY 2, PER1, and PER2, regulating their nuclear translocation and proteosomal degradation (38–41). Mutations in the phosphorylation sites of PER, for example, can either shorten or lengthen the cycle dramatically due to altered accessibility of the protein to degradation (39). Furthermore, alterations of the PER phosphorylases CK1 ϵ and CK1 δ can have a similar effect (42–44): a gain of function mutation of CK1 δ, which leads to a more effective phosphorylation and hence faster degradation of PER, causes familial cases of ASPS (28). Apart from its direct mutagenic properties, genotoxic stress in general is also known to influence the length of tau: the clock proteins CRY1 and CRY2 are ontogenetically derived from DNA repair enzymes and upregulated in response to mutation accumulating in the cells (45). Both CRYs are repressors of clock gene transcription and thus lengthen and stabilize the intrinsic SCN rhythm (46–48). The more potent action of CRY1 in slowing the clock is also attenuated by CRY2, demonstrating separate roles of the two proteins within the core clock mechanism (48).

The amplitude of circadian oscillation as another discrete clock property is associated with the vulnerability of the system to resetting stimuli in a directly proportional manner (6, 33). A mutation of CLOCK in mice has been shown to dampen the amplitude of the circadian oscillation, causing phenotypes to exhibit a greater susceptibility to light as a zeitgeber than wild type (49). Furthermore, neuropeptides regulating the internal synchronization of the SCN neurons and alterations of upstream (50, 51) and downstream mechanisms (52) seem to be involved. In humans, the amplitude of the oscillation shows considerable interindividual differences as well (33). Accordingly, our patients’ reluctance to various and even combined attempts to shift the circadian phase could be an expression of a comparably strong circadian oscillation, independent of the extreme cycle length and thus explaining the observed treatment failure.

Are there other options left to achieve entrainment? First of all, headaches caused by melatonin administration are a common side effect, which habituate during the course of the treatment (53). Therefore, it could be a justified to wait for the headaches to subside, especially since some case studies report a delayed treatment response to melatonin (54). Additionally, the use of more MT2-selective melatonin receptor agonists (20) could prove superior in the phase-shifting abilities compared to melatonin, with a theoretically reduced propensity to cause side effects (55, 56). Second, light therapy was conducted for only 30 min in the morning – a longer, more intense therapy could have had the desired effect, particularly when one assumes that our patient expressed a comparatively strong circadian rhythm. Finally, based on recent research which points to a greater plasticity of the cycle length than previously thought (57), the third possibility would be to gradually change the period length in a laboratory setting and slowly entrain it to a ~24-h LD cycle.

The exact cause of the N24HSWD in the presented case remains hard to pinpoint and subjects to speculation. However, considering the physiology of the circadian cycle, the complexity of steps, and the influencing factors involved, a single cause seems questionable. The conclusion for treatment options in this case and in general must therefore be to apply a multimodal therapy concept with a combination of the above-mentioned strategies adapted to the most likely causing factor. Nevertheless, randomized controlled trials studying the optimal combination of the established strategies and incorporation of new therapeutic approaches would be vital to finally establish a treatment consensus for N24HSWD in sighted patients.

## Ethics Statement

This report describes the clinical case of a patient who was referred to our centre for routinary clinical evaluation. The patient consented to all analyses conducted and to publication of the case history and test results.

## Author Contributions

Conceived and designed the experiments: VB and CC; performed the experiments: CG, VB, FM and AE; analyzed the data: VB, CG, CC, FM, and AE; and contributed to the writing of the manuscript: SH, CG, CC, VB and DB.

## Conflict of Interest Statement

The authors declare that the research was conducted in the absence of any commercial or financial relationships that could be construed as a potential conflict of interest.
